# Interstitial Injection of Carbolic Acid in Piles

**Published:** 1878-05

**Authors:** 


Interstitial Injection of Carbolic Acid in Piles.—Injection of piles is now becoming a very popular method of treating this painful and troublesome affection. From personal observation I am satisfied that the method deserves all its popularity, for it is comparatively easy to practice by the use of an ordinary hypodermic syringe, gives but little pain, and is far superior to any other method hitherto adopted. Such is the testimony of many eminent surgeons and a large number of respectable practitioners who have given it a fair trial.

The acid is not used full strength, though it might be with good results, but is diluted one-third to one-half or more with water or glycerine, or the two combined. My own method has been to mix equal parts of the strong acid in solution, water and glycerine, to which I add a small quantity of tannin and morphia. The amount of this to be used varies from three to fifteen drops, according to the size of the tumor to be operated upon. About the only preparation usually necessary to be made (or care to observe) is to evacuate the bowels well just before the operation, and to remain quiet, and avoid solid aliments for a few days afterwards.

A single injection is most usually sufficient to effect the removal of an ordinary-sized tumor, but where the tumor is very large or lobulated, it may require two or more injections; and where several separate tumors exist, they will each require an injection.

I have always been highly gratified by the rapidity of the favorable results following this plan of treatment, and although it may not be absolutely safe in all cases, I have not yet seen or heard of it producing any serious effects, and I always resort to it with the strongest confidence of a favorable result. Who discovered the method, or rather brought it to the notice of the profession, I do not know, but I have seen notices of it in several journals not long since. It appears to have been practiced for some time by a class of quacks, before it obtained much favor with the regular profession, thus illustrating the fact that the profession is not always first to adopt a good thing, notwithstanding their untiring researches and efforts to relieve and palliate the ills of the human race.

In pile tumors of moderate size and purely external, I do not think that the interstitial injection of carbolic acid has any advantages over the bistoury, for in such cases a simple incision is altogether satisfactory.

